# Laughter and Stress Relief in Cancer Patients: A Pilot Study

**DOI:** 10.1155/2015/864739

**Published:** 2015-05-24

**Authors:** S. H. Kim, Y. H. Kim, H. J. Kim

**Affiliations:** ^1^Department of Nursing, ASAN Medical Center, Seoul 138-736, Republic of Korea; ^2^Department of Preventive Medicine, University of Ulsan College of Medicine, 43-Gil Olympic-ro, Songpa-gu, Seoul 138-736, Republic of Korea

## Abstract

The purpose of this study was to examine the effect of a therapeutic laughter program and the number of program sessions on anxiety, depression, and stress in breast cancer patients. A randomized controlled trial was conducted involving 31 patients who received four sessions of therapeutic laughter program comprised and 29 who were assigned to the no-program control group. Scores for anxiety, depression, and stress were measured using an 11-point numerical rating scale. While no change was detected in the control group, the program group reported reductions of 1.94, 1.84, and 2.06 points for anxiety, depression, and stress, respectively (*p* < 0.01, *p* < 0.01, and *p* < 0.01). Scores decreased significantly after the first therapeutic laughter session (*p* < 0.05, *p* < 0.01, and *p* < 0.01). As the therapeutic laughter program was effective after only a single session in reducing anxiety, depression, and stress in breast cancer patients, it could be recommended as a first-line complementary/alternative therapy.

## 1. Introduction

The increase in the survival of cancer patients has created interest in quality of life and the various factors affecting it. Breast cancer is the second most frequent cancer in Korean women and occurs most often in middle-aged women in their 40s and 50s [1]. The 5-year survival rate of breast cancer patients has continued to increase since 1993 and it has the second highest survival rate at 91.3% in the last 5 years, beaten only by thyroid cancer [1]. Among the various factors reported to affect quality of life is psychological stress, which leads to depression and anxiety [2]. Psychological stress prevalence is very high among breast cancer patients in Korea, with depression affecting 44% of the cancer patient population [3] and 36% of breast cancer patients undergoing radiation therapy; meanwhile, 21% experience anxiety [4]. Therefore, effective interventions to reduce psychological stress are needed urgently.

Laughter is regarded as a long-standing complementary and alternative therapy since 1970 [5]. As laughter is a noninvasive complementary/alternative therapy, the use of laughter therapy has spread rapidly. Currently, there are several laughter therapy clubs around the world in which people gather to practice laughter and laughing on purpose; this fake laughter gradually becomes effective in releasing “anti-stress and joyful hormones” [6]. Studies on the effect of laughter have been actively promoted, and some studies have specifically targeted dialysis patients, elderly people, transplant patients, postpartum women, and smokers [6–9].

Studies have found a variety of positive effects of laughter therapy on anxiety, depression, tension, rage, and general health, and it has been found useful for insomnia, pain relief, improving pulmonary function, and increasing immunity [6, 7, 10–15]. Meanwhile, some studies have been conducted among cancer patients with laughter therapy demonstrating major positive effects on quality of life, resilience, immunity, anxiety, depression, and stress [14, 16–18]. Nonetheless, the therapeutic effects of laughter for cancer patients are not at a point where they can be confirmed because most previous research was conducted using comparisons without randomization [10–13]. Moreover, a standardized therapeutic laughter program (TLP) has not been developed yet, which means that laughter therapy has not been actively promoted by the medical community for cancer patients. While the results of TLP for health have been positive [19], and there is an “abundance of non-evidence-based opinion” regarding TLP in the literature, so an evidence-based approach is required [20].

In order to evaluate the effects of laughter for cancer patients accurately, various sources of bias should be controlled thoroughly. Thus, we designed a randomized controlled trial to compare the effect of a TLP consisting of four sessions and significant decrease of depression, anxiety, and stress was reported among breast cancer patients undergoing radiotherapy [16]. Data collected through conduction of this trial form the basis of our comparative analysis. The secondary purpose of this study was to measure whether the effects of laughter therapy differ based on the number of sessions attended. An additional concern was that measuring psychological stress routinely using multiple item questionnaires such as the Hospital Anxiety Depression Scale (HADS) [21] and Brief Encounter Psychosocial Instrument-Korean version (BEPSI-K) [22] is difficult in a busy outpatient setting. Therefore, we evaluated the level of depression, anxiety, and stress additionally using a single-question, 11-point numerical rating scale (NRS).

## 2. Methods

This study was conducted as a secondary analysis using data collected from a randomized controlled trial to investigate the effects of laughter on depression, anxiety, and stress among breast cancer patients in comparison to a nontreatment control group [16]. This study was approved by the ASAN Medical Center Institutional Review Board.

### 2.1. Participants and Intervention

Breast cancer patients receiving postoperative radiation therapy were recruited between September and October 2008 at the ASAN Medical Center. We excluded patients with psychiatric problems, including major depressive disorder and anxiety disorder, as determined by medical record review; each participant was interviewed and checked for their past or current medical problem, including psychiatric problems, during their admission before operation, and this was reaffirmed via the self-reporting questionnaire on their baseline visit before randomization.

A total of 62 patients were randomized into the TLP and nonintervention groups. The TLP was administered for four sessions by a licensed TLP trainer, with each session lasting 60 minutes; the control group did not receive any intervention. The program consisted of periods of loud, prolonged laughter together with information about the effects of the TLP (details of the TLP are described in Supplement 1 in Supplementary Material available online at http://dx.doi.org/10.1155/2015/864739).

### 2.2. Data Collection and Statistical Analysis

**Figure 1:**
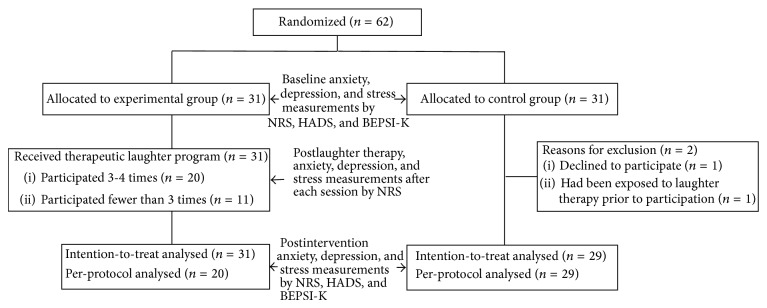
Flow chart of the randomized controlled trial to evaluate antipsychological stress effects of laughter therapy in breast cancer patients. NRS (numerical rating scale); HADS (Hospital Anxiety Depression Scale); BEPSI-K (Brief Encounter Psychosocial Instrument-Korean version).

To investigate the effects of the TLP, a single-question, 11-point (ranging from 0 to 10) NRS was used to measure anxiety, depression, and stress levels (details of the NRS are described in Supplement 2); as gold standards, anxiety and depression were measured using the HADS [21], and stress was measured using the BEPSI-K [22]. The TLP group participants and the control participants were measured for anxiety, depression, and stress before and after participation in four TLP sessions using the NRS, HADS, and BEPSI-K; in addition, the NRS scores of the TLP group for anxiety, depression, and stress were measured after each TLP session (Figure 1).

In order to confirm the validity of the NRS, the correlations between it and the HADS and BEPSI-K were analyzed using Spearman's rank correlation coefficient test. Baseline NRS scores for anxiety, depression, and stress before the TLP were compared using independent *t*-tests and the Mann-Whitney *U* test. The differences between the NRS scores for anxiety, depression, and stress in the two groups were examined using an analysis of covariance, adjusted for baseline stress scores and marital status, which had a significant effect in a previous study [16]. The primary analysis was an intention-to-treat analysis (ITT), and a per-protocol analysis (PP) was performed as a secondary analysis. Only patients who participated in more than two of the four TLP sessions were included in the PP analysis. The effects of the number of TLP sessions attended were analyzed using a repeated measures analysis of variance with Bonferroni's posttest correction. All analyses were performed using SPSS software (version 18.0).

## 3. Results

Of the 60 patients who participated in the study, 29 in the control group and 31 in the TLP group were included in the ITT analysis (Figure 1); for the PP analysis, 20 from the TLP group who had more than two sessions and 29 in the control group were included. There was no significant difference except for marital status between the general characteristics or disease characteristics of the two groups (Table 1). Spearman's rank correlation between the NRS and HADS scores for anxiety is 0.59 (*p* < 0.01) and, for depression, 0.62 (*p* < 0.01). The correlation between the NRS and BEPSI-K for stress is 0.63 (*p* < 0.01). As evaluated, the NRS scores for anxiety displayed a moderate correlation with the HADS scores. For depression and stress, the NRS scores had a strong correlation with the HADS and BEPSI-K scores.

The NRS scores for anxiety, depression, and stress of the TLP group decreased by 1.94, 1.84, and 2.06 points, respectively, whereas no changes were reported in the control group (*p* < 0.01, *p* < 0.01, and *p* < 0.01) (Table 2). In the PP analysis, there were also significant differences in the NRS scores for anxiety, depression, and stress between the TLP group and control group (Table 2).

**Figure 2:**
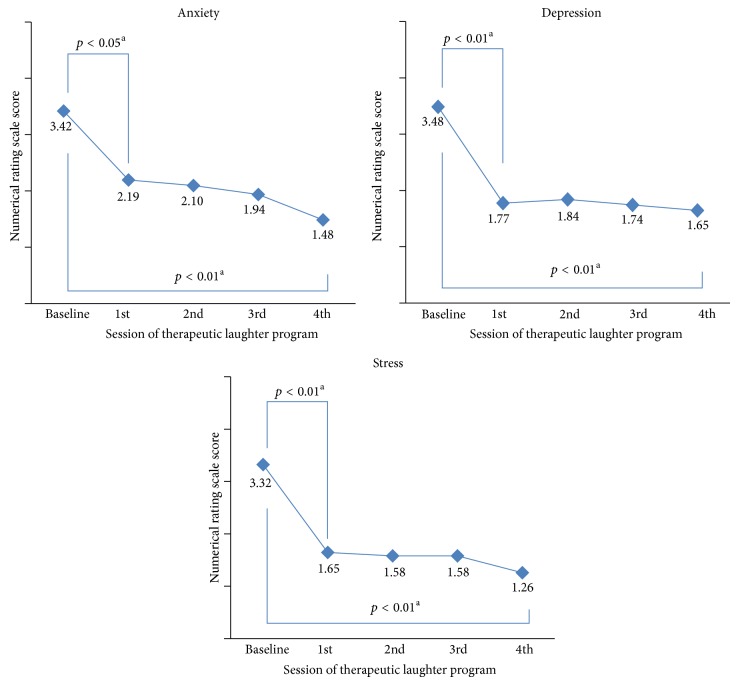
Anxiety, depression, and stress scores of the experimental group according to attended numbers of laughter therapy sessions. *p* values were calculated by (a) repeated measures analysis of variance test.

The results obtained by repeatedly measuring and comparing the NRS scores for anxiety, depression, and stress of the TLP group showed that, as the number of TLP sessions attended increased, the NRS scores for anxiety, depression, and stress decreased significantly (*p* < 0.01, *p* < 0.01, and *p* < 0.01) (Figure 2). The posttest results after every TLP session showed that anxiety, depression, and stress levels decreased (*p* < 0.05, *p* < 0.01, and *p* < 0.01) between the presession scores and the first postsession scores, and there were no further significant changes (Figure 2).

## 4. Discussion

TLP was effective in lowering anxiety, depression, and stress as measured by the NRS in breast cancer patients undergoing radiation treatment in comparison to the control group. This finding concurs with the HADS and BEPSI-K results [16]. Therefore, using the NRS instead of the HADS (14 items) and BEPSI-K (5 items) could be a useful option among routine repeated measures in busy outpatient settings.

Moreover, we found that the anxiety, depression, and stress levels of breast cancer patients could be reduced after a single TLP session. The reduction of anxiety, depression, and stress after TLP has been reported in a number of studies [6, 8, 23–26], and various physiological effects of TLP have also been reported [8, 27–30]. Although the mechanism of the TLP's effect is not well understood, researchers have reported that laughing reduces neuroendocrine and stress-related hormones, and a hypothesis regarding the TLP mechanism that contributes to psychological stress reduction has been suggested [27]. We found that the antiphysiological effects were evident after one session of TLP. However, while we reported that a single session of TLP was effective in nurses [31], another study conducted among the elderly found that a single session was not effective for depression and anxiety [32].

These conflicting findings may be due to the difference between the study populations. While the study on nurses was comprised of participants who were young and female, the elderly participants in the second study included men and women who were over 60. In the present study, the participants consisted only of female breast cancer patients in their 40s. Therefore, the homogeneity of the participants should be considered when planning laughter programs. In this study, the TLP consisted of a detailed standardized program to make participants laugh a great deal. It comprised activities that induced loud laughter as a result of direct participation and physical activity appropriate to the characteristics and age of the breast cancer patients. This is thought to have contributed to enhancing the TLP effects.

Moreover, across the studies, the number and duration of TLP sessions varied so that the TLPs comprised of eight 20-minute sessions or a single 60-minute session showed improved mood states [6, 16–18, 23–25]. Although a single-session TLP could be effective as reported in our study, we could not assert that the effect would last for a clinically meaningful period. Because the TLP was implemented four times, repeated participation in the TLP could affect the maintenance of antiphysiological effects. As this study was only a “pilot study” conducted to measure the immediate antistress effects of TLP, long-term effects could not be measured. This means that the effects of the TLP cannot be compared with the effect of this intervention in persons with a full-blown psychiatric disease. Moreover, the highly selective inclusion and exclusion criteria could limit the generalizability of the results. Because of these limitations, further studies should assess the long-term effects of TLP and find a mechanism through which laughter could affect mental health.

## 5. Conclusion

The TLP is effective in reducing anxiety, depression, and stress in breast cancer patients, and such effects can be attained after only one session. This study is useful as there has been little previous analysis of the effect of the number of TLP sessions. TLP could also be used effectively in clinical practice settings, as it is a noninvasive, easy-to-use complementary/alternative therapy; therefore, it is recommended that medical professionals use a standardized TLP as a complementary intervention to assist with patient treatment.

## Supplementary Material

Supplementary 1: The program consisted of periods of loud, prolonged laughter together with information about the effects of the TLP. Starting with an brief introduction including the positive effect of the laughter, participants were lead to various type of laughing (e.g. laughing in rhythm with clapping, laughing for a long time, laughing with the whole body, laughing in various ways, and laughing together with dance routines). After the session ended up with calming the mind and emotion sharing
Supplementary 2: Descriptions on the NRS are sufficient as in the manuscript.

## Figures and Tables

**Table 1:** Baseline characteristics of intention-to-treat population of two groups.

Characteristics	Subtotal	Experimental group (*n* = 31)	Control group (*n* = 29)	*p* value
		*n* (%)	*n* (%)	
Age (years)				
<40	13	9 (29%)	4 (14%)	0.38^b^
40–49	23	12 (39%)	11 (38%)	
50–59	17	8 (26%)	9 (31%)	
≥60	6	2 (6%)	5 (17%)	
Marital status				
Yes	50	22 (71%)	28 (97%)	0.01^b^
No	10	9 (29%)	1 (3%)	
Education level				
≤Middle school	8	3 (38%)	5 (63%)	0.35^a^
High school	24	15 (63%)	9 (38%)	
≥College	28	13 (46%)	15 (54%)	
Cancer stage				
0	4	2 (7%)	2 (7%)	0.83^b^
I	25	11 (36%)	14 (48%)	
II	21	12 (39%)	9 (31%)	
III	10	6 (19%)	4 (14%)	
Operation				
Breast conserving operation	53	28 (90%)	25 (86%)	0.70^b^
Mastectomy	7	3 (10%)	4 (14%)	
Past treatment				
Operation	29	18 (29%)	11 (38%)	0.12^a^
Operation and chemotherapy	31	13 (71%)	18 (62%)	
Current cotreatment				
None	14	9 (29%)	5 (17%)	0.28^b^
Hormone therapy	46	22 (71%)	24 (83%)	

*p* values were calculated by (a) Chi^2^ test and (b) Fisher's exact test.

**Table 2:** Change of numerical rating scale scores for anxiety, depression, and stress after therapeutic laughter program.

	Intention-to-treat analysis			Per-protocol analysis		
	Experimental group (*n* = 31)	Control group (*n* = 29)	*p* value	Experimental group (*n* = 31)	Control group (*n* = 29)	*p* value
	Mean ± SD	Mean ± SD		Mean ± SD	Mean ± SD	
Baseline						
Anxiety	3.42 ± 2.08	3.21 ± 2.24	0.66^b^	3.20 ± 2.31	2.97 ± 1.92	0.70^a^
Depression	3.48 ± 2.14	2.97 ± 1.92	0.33^a^	3.30 ± 2.03	3.21 ± 2.24	0.83^b^
Stress	3.32 ± 2.09	3.62 ± 1.95	0.57^a^	3.35 ± 2.21	3.62 ± 1.95	0.38^b^
Postintervention						
Anxiety	1.48 ± 1.46	3.31 ± 2.22	<0.01^c^	1.10 ± 1.41	3.31 ± 2.22	<0.01^c^
Depression	1.65 ± 1.62	3.31 ± 2.04	<0.01^c^	1.30 ± 1.66	3.31 ± 2.04	<0.01^c^
Stress	1.26 ± 1.32	3.72 ± 1.81	<0.01^c^	0.80 ± 1.20	3.72 ± 1.81	<0.01^c^
Difference						
Anxiety	−1.94 ± 1.97	0.10 ± 1.97	<0.01^b^	−2.20 ± 1.85	0.10 ± 1.97	<0.01^b^
Depression	−1.84 ± 1.63	0.34 ± 1.97	<0.01^b^	−1.90 ± 1.55	0.34 ± 1.97	<0.01^b^
Stress	−2.06 ± 2.00	0.10 ± 2.14	<0.01^b^	−2.55 ± 1.88	0.10 ± 2.14	<0.01^b^

*p* values were calculated by (a) independent *t*-test, (b) Mann-Whitney test, and (c) analysis of covariance test adjusting for marital status and baseline distress score.

## References

[1] Jung K.-W., Won Y.-J., Kong H.-J., Oh C.-M., Lee D. H., Lee J. S. (2014). Cancer statistics in Korea: incidence, mortality, survival and prevalence in 2011. *Cancer Research and Treatment*.

[2] Byun H. S., Kim G. D. (2012). Impacts of fatigue, pain, anxiety, and depression on the quality of life in patients with breast cancer. *Asian Oncology Nursing*.

[3] Park B.-W., Sook Y. H. (2009). Depression and coping in breast cancer patients. *Journal of Breast Cancer*.

[4] So W. K. W., Marsh G., Ling W. M. (2009). The symptom cluster of fatigue, pain, anxiety, and depression and the effect on the quality of life of women receiving treatment for breast cancer: a multicenter study. *Oncology Nursing Forum*.

[5] Cousins N. (1976). Anatomy of an illness (as perceived by the patient). *The New England Journal of Medicine*.

[6] Ghodsbin F., Ahmadi Z. S., Jahanbin I., Sharif F. (2015). The effects of laughter therapy on general health of elderly people referring to jahandidegan community center in Shiraz, Iran, 2014: a randomized controlled trial. *International Journal of Community Based Nursing and Midwifery*.

[7] Bennett P. N., Parsons T., Ben-Moshe R. (2014). Laughter and humor therapy in dialysis. *Seminars in Dialysis*.

[8] Dolgoff-Kaspar R., Baldwin A., Scott Johnson M., Edling N., Sethi G. K. (2012). Effect of laughter yoga on mood and heart rate variability in patients awaiting organ transplantation: a pilot study. *Alternative Therapies in Health and Medicine*.

[9] Shin H. S., Ryu K. H., Song Y. A. (2011). Effects of laughter therapy on postpartum fatigue and stress responses of postpartum women. *Journal of Korean Academy of Nursing*.

[10] Bennett M. P., Lengacher C. A. (2006). Humor and laughter may influence health. I. History and background. *Evidence-based Complementary and Alternative Medicine*.

[11] Bennett M. P., Lengacher C. (2006). Humor and laughter may influence health: II. Complementary therapies and humor in a clinical population. *Evidence-Based Complementary and Alternative Medicine*.

[12] Bennett M. P., Lengacher C. (2008). Humor and laughter may influence health: III. Laughter and health outcomes. *Evidence-Based Complementary and Alternative Medicine*.

[13] Bennett M. P., Lengacher C. (2009). Humor and laughter may influence health IV. humor and immune function. *Evidence-Based Complementary and Alternative Medicine*.

[14] Cho E. A., Oh H. E. (2011). Effects of laughter therapy on depression, quality of life, resilience and immune responses in breast cancer survivors. *Journal of Korean Academy of Nursing*.

[15] Christie W., Moore C. (2005). The impact of humor on patients with cancer. *Clinical Journal of Oncology Nursing*.

[16] Kim S. H., Kim Y. H., Kim H. J., Lee S. H., Yu S. O. (2009). The effect of laughter therapy on depression, anxiety, and stress in patients with breast cancer undergoing radiotherapy. *Asian Oncology Nursing*.

[17] Choi J., Kim K., Cha S., Pyo H., Kim Y. (2010). Effects of laughter therapy on mood, pain, and stress of mastectomy patients. *Journal of Korean Clinical Nursing Research*.

[18] Han H. J., Park A., Kim H. S., Moon H., Park Y. (2011). The effects of laughter therapy on stress responses in patients with preoperative breast cancer. *Journal of Korean Oncology Nursing*.

[19] Strean W. B. (2009). Laughter prescription. *Canadian Family Physician Médecin de Famille Canadien*.

[20] McCreaddie M., Wiggins S. (2008). The purpose and function of humour in health, health care and nursing: a narrative review. *Journal of Advanced Nursing*.

[21] Zigmond A. S., Snaith R. P. (1983). The hospital anxiety and depression scale. *Acta Psychiatrica Scandinavica*.

[22] Frank S. H., Zyzanski S. J. (1988). Stress in the clinical setting: the brief encounter psychosocial instrument. *The Journal of Family Practice*.

[23] Yu J. A., Kim K. S. (2009). Effects of laughter therapy on stress response and pain of military personnel with low back pain in hospital. *Journal of Muscle and Joint Health*.

[24] Lee K. I., Eun Y. (2010). Effect of laughter therapy on pain, depression and sleep with elderly patients in long term care facility. *Journal of Muscle and Joint Health*.

[25] Shin B. J., Kim H. S., Lee M. H. (2010). Effect of humorous video tape on depression and stress response in patients undergoing hemodialysis. *Journal of East-West Nursing Research*.

[26] Ko H.-J., Youn C.-H. (2011). Effects of laughter therapy on depression, cognition and sleep among the community-dwelling elderly. *Geriatrics & Gerontology International*.

[27] Berk L. S., Tan S. A., Fry W. F. (1989). Neuroendocrine and stress hormone changes during mirthful laughter. *American Journal of the Medical Sciences*.

[28] Dillon K. M., Minchoff B., Baker K. H. (1985). Positive emotional states and enhancement of the immune system. *International Journal of Psychiatry in Medicine*.

[29] Hayashi K., Hayashi T., Iwanaga S. (2003). Laughter lowered the increase in postprandial blood glucose. *Diabetes Care*.

[30] Takahashi K., Iwase M., Yamashita K. (2001). The elevation of natural killer cell activity induced by laughter in a crossover designed study. *International Journal of Molecular Medicine*.

[31] Park M. K. (2010). *The Effect of One Time Laughter Therapy on Stress of ICU Nurses*.

[32] Kim Y. S., Jun S. S. (2009). The influence of one-time laughter therapy on stress response in the elderly. *Journal of Korean Academy Psychiatric and Mental Health Nursing*.

