# Supplementary material for: Laughter and Stress Relief in Cancer Patients: A Pilot Study
Source: Evid Based Complement Alternat Med. 2015 May 24;2015:864739. doi: 10.1155/2015/864739 (PMC4439472; doi:10.1155/2015/864739)
Supplement: Supplementary file 1 — Supplementary 1: The program consisted of periods of loud, prolonged laughter together with information about the effects of the TLP. Starting with an brief introduction including the positive effect of the laughter, participants were lead to various type of laughing (e.g. laughing in rhythm with clapping, laughing for a long time, laughing with the whole body, laughing in various ways, and laughing together with dance routines). After the session ended up with calming the mind and emotion sharing Supplementary 2: Descriptions on the NRS are sufficient as in the manuscript. [file 864739.f1.pdf]

Supplement1. Details of the therapeutic laughter program

Section	Content
Intro (10 mins)	**Ice breaking**<br>Introducing oneself to other participants<br>**Introduction of the program**<br>Introduction on the schedule of laughter therapy session<br>Brief information on the positive effect of the laughter
Main body (40 mins)	**Warming up**<br>Relaxation of body muscles<br>Relaxation of facial muscles used in laughing<br>**Laughing in rhythm with clapping**<br>Health-claps; Clap to stimulate the reflex points on the palm by using the fingers, the palms and the back of the hands<br>**Laughing for a long time**<br>Laugh loudly together for 5 seconds, 10 seconds, and 15 seconds<br>**Laughing with the whole body**<br>Clap hands while laughing<br>Clap hands and stomp feet while laughing<br>Clap hands, stomp feet, and shake head while laughing<br>Clap hands, stomp feet, and shake the whole body while laughing<br>**Laughing in various ways**<br>Snow White laugh; Pretend of the palm as a mirror and laugh -'Mirror, mirror, on the wall, who is the prettiest of them all?'/ 'That would be me, HAHAHA' -while looking into the palm<br>Cell phone laugh; Pretend to receive a call while walking on the street and laugh by oneself<br>Lion laugh; Laugh while imitating lions' actions and facial expressions<br>Celeb laugh; Imitate laughs that are specific to certain celebrities<br>**Laughing together with dance routines**<br>Dance along to the music, and begin laughing in various ways (HAHAHA, HOHOHO, HIHIHI)
Wrap up (10 mins)	Calm people's minds by playing a relaxing music or reading out a good writing (poem).<br>Share what one felt by participating in laughing therapy

SUPPLEMENT2. Numerical rating scale for anxiety, depression and stress levels

● Please indicate the number 0(lowest) to 10(highest) that best describe how much depression you have been in today.

0	1	2	3	4	5	6	7	8	9	10

No depression

Worst depression imaginable

● Please indicate the number 0(lowest) to 10(highest) that best describe how much anxiety you have been in today.

0	1	2	3	4	5	6	7	8	9	10

No anxiety

Worst anxiety imaginable

● Please indicate the number 0(lowest) to 10(highest) that best describe how much stress you have been in today.

0	1	2	3	4	5	6	7	8	9	10

No stress

Worst stress imaginable
